# Effect of Rejuvenator Containing Dodecyl Benzene Sulfonic Acid (DBSA) on Physical Properties, Chemical Components, Colloidal Structure and Micro-Morphology of Aged Bitumen

**DOI:** 10.3390/ma11081476

**Published:** 2018-08-20

**Authors:** Dongliang Kuang, Zhou Ye, Lifeng Yang, Ning Liu, Zaihong Lu, Huaxin Chen

**Affiliations:** 1 Engineering Research Central of Pavement Materials, Ministry of Education, Chang’an University, Xi’an 710064, China; joe_yezhou@163.com; 2 School of Civil Engineering, Qinghai University, Xining 810016, China; qhyanglifeng@126.com; 3 Highway Researching and Designing Institute of Qinghai Province, Xining 810001, China; 1969ln@163.com; 4 Inner Mongolia Transportation Design Institute Co. LTD., Huhhot 010000, China; luzaihongnm@163.com

**Keywords:** aged bitumen, rejuvenator, solubilizer, colloidal structure, micro-morphology

## Abstract

DBSA was used as a solubilizer together with conventional rejuvenator (CR) to produce a solubilized rejuvenator (SR), two kinds of aged bitumen involving TFOT aged bitumen and PAV aged bitumen were obtained by thin film oven test (TFOT) and pressurized aging vessel (PAV), respectively. Effects of CR and SR on the physical properties, chemical components, colloidal structure and micro-morphology of TFOT aged bitumen and PAV aged bitumen were investigated. Testing results of physical properties and chemical components indicated that CR and SR can replenish aged bitumen with necessary aromatics, TFOT aged bitumen that chemical component variation deteriorates its physical properties. With regard to PAV aged bitumen, of which the performance attenuation lies in chemical components variation and colloidal structure transformation, even if the content of CR reached up to 10 wt %, the regenerated bitumen cannot meet the regeneration requirement yet due to its definite influence on colloidal structure transformation, comparatively, sulfonic group in SR can react with the superficial atoms of asphaltenes to reform a solvation layer to facilitate the colloidal structure transformation of PAV aged bitumen, performance and beelike structure of regenerated PAV aged with bitumen with 10 wt % SR were approximated to that of virgin bitumen.

## 1. Introduction

Bitumen has been widely applied in highway construction due to its excellent pavement performance [1,2]. As an organic binder, bitumen is subjected to aging as a consequence of heat and oxygen during storage, mixing, transportation and paving, as well as service period [3,4,5], which reduces the performance and service time of asphalt pavement [6,7]. Maintenances and reconstructions are practically necessary for the damaged asphalt pavement to dispose of the premature distresses [8]. In these processes, damaged asphalt pavement is firstly smashed and collected by road milling machine, which will produce huge amounts of reclaimed asphalt pavement (RAP) [9]. For the sake of resource conservation and environmental protection, recycling of RAP is an effective and sustainable technique to cope with the RAP [10]. Compared with virgin asphalt mixture, RAP has lower road performance due to bitumen aging [11], therefore the regenerating of aged bitumen is important for the recycling of RAP [12].

It has been reported in previous studies that aging of bitumen is the main factor resulting in the premature failure of asphalt pavement, which can be explained as following: The chemical components proportions of bitumen are changed by aging [13,14], namely the asphaltenes content increases and aromatics content declines, which result in the decreasing in penetration and ductility as well as increasing in softening point and viscosity [15].

Aging not only damage the performance, but also affects the colloidal structure involving the type of colloidal structure and compatibility between chemical components. As it is known, the colloidal solution of bitumen is composed of asphaltenes covered by solvation layer acting as the micellar nucleus and malthene acting as the dispersing medium [16]. However, the solvation layer is subjected to be destroyed by aging, and then the asphaltenes will precipitate from the other chemical components [17], coupling with the increment in asphaltenes content induced by aging, the surface of bitumen becomes rough, which can be observed by Atomic Force Microscopy (AFM) [18]. The AFM testing conducted on bitumen indicates that the bitumen exhibits bee-like structure [19], and the dimension of bee-like structures of bitumen as well as the roughness of bitumen surface are increased after aging [20].

The aged bitumen can be classified as mild aged bitumen and severe aged bitumen according to the residual penetration of aged bitumen [21]. For mild aged bitumen, its chemical components variation after aging dominantly influences the performance attenuation of bitumen, thus it can be effectively regenerated by replenishing with necessary amount of aromatics, however, with regard to severe aged bitumen, its performance attenuation is attributed to both chemical components variation and colloidal structure transformation [22], therefore replenishing aromatics and recovering colloidal structure are both necessary for regeneration of severe aged bitumen.

Rejuvenators consisting of high proportion of aromatics have been widely applied for recycling of RAP [23], which can replenish aged bitumen with necessary aromatics, thus it can regenerate mild aged bitumen effectively. However, for severely aged bitumen, of which the colloidal structure has changed, the regeneration effect of rejuvenators including high proportion of aromatics is definite due to its little influence on recovering of colloidal structure [24].

Dodecyl benzene sulfonic acid (DBSA) as a surfactant has been widely used in synthesis of detergent. The sulfonic group in DBSA can react with superficial atoms of removed substance by hydrogen-bond interaction and reform a spatial stability layer to facilitate the dissolving of removed substance [25]. In this paper, the DBSA was used as a solubilizer together with conventional rejuvenator (CR) to produce a solubilized rejuvenator (SR). Effects of DBSA on the physical properties, chemical components, colloidal structure and micro-morphology of different aged bitumen were investigated.

## 2. Materials and Methods

### 2.1. Materials

Bitumen, with penetration of 70 grade, was supplied by Shaanxi Guolin Hi-Tech Material Co., Ltd., Xi’an, China, its physical properties and chemical component involving saturates (Sa), aromatics (Ar), resin (Re) and asphaltenes (As), were listed in Table 1. Fluid catalytic cracking slurry (FCC) heated to 100 °C and bitumen with penetration of 70 grade heated to 130 °C was initially blended in an iron container (Shaanxi Jingbo Hi-Tech Co., Ltd., Xi’an, China) for 15 min at a speed of 500 rpm to produce conventional rejuvenator (CR). For preparation of solubilized rejuvenator (SR), 1.5 wt % DBSA by weight of CR heated to 60 °C was added into CR heated to 110 °C and blended for 15 min at a speed of 500 rpm. The physical properties and chemical components of SR and CR are listed in Table 2.

### 2.2. Preparation of Aged Bitumen

Two aged bitumens were obtained by thin film oven test (TFOT, aging at 163 °C for 5 h according to [27] and pressurized aging vessel (Prentex, San Francisco, CA, USA) (PAV, aging at 100 °C for 20 h under 2.1 MPa of air according to [28], respectively. The TFOT was employed to simulate the short-term aging including storage, transport, mixing and paving process of the bitumen, whereas the PAV was used to simulate the long-term aging of the bitumen in service period. The aged bitumen prepared by TFOT and PAV were denoted as TA and PA respectively.

### 2.3. Preparation of Regenerated Bitumen

SR (or CR) heated to 110 °C was firstly added into aged bitumen heated to 150 °C, and the blended at 150 °C with a speed of 500 rpm for 30 min to prepare regenerated bitumen, the content of SR (or CR) were 2, 4, 6, 8 and 10 wt % by weight of aged bitumen. The regenerated TA containing SR and CR were denoted as SR-TA and CR-TA respectively; and the regenerated PA containing SR and CR were denoted as SR-PA and CR-PA respectively.

### 2.4. Physical Properties Testing

Physical properties of virgin, aged, and regenerated bitumen, including softening point, penetration at 25 °C, ductility at 15 °C, and viscosity at 135 °C were tested according to the methods in [29], [30], [26] and [31], respectively.

### 2.5. Chemical Components Testing

Bitumen was dissolved in n-heptane (Shaanxi Jingbo Hi-Tech Co., Ltd., Xi’an, China), asphaltenes was firstly separated according to [32] and its content was determined. Residual n-heptane was evaporated and the residues were dissolved in toluene (Shaanxi Jingbo Hi-Tech Co., Ltd., Xi’an, China) to form a solution of 2% (w/v), then 1 µL sample solution was spotted on chromarods using a spotter. Afterwards, the chromarods were expanded in n-heptane and toluene respectively. The contents of saturates, aromatics and resins were determined using Iatroscan MK-6 analyzer (Iatro Laboratories Inc., Tokyo, Japan).

### 2.6. Colloidal Structure Characterization

The colloidal structure can be characterized by Penetration Index (PI) [26] and Colloidal Stability Index (CSI) [24]. The CSI and CSI are used for characterization of colloidal structure type and colloidal structure stability, respectively.

With respect to the PI of less than −2, −2 to 2 and more than 2, the corresponding colloidal structure of bitumen are Sol, Sol-Gel and Gel respectively [16]. The PI of virgin, aged, and regenerated bitumen were calculated according to [26] in this paper.

The CSI calculated based on the chemical components testing [24], is adapted to characterize the stability of colloidal structure of regenerated bitumen in this paper, the more the CSI of regenerated bitumen approximates that of virgin bitumen, the more the aged bitumen is regenerated. For obtaining the CSI, the chemical components of the samples were determined according to the methods mentioned in 2.5, and then the CSI were calculated according to the following equation:

(1) $$CSI=\frac{ArC+ReC}{AsC+SaC}$$

where the *ArC*, *ReC*, *AsC* and *SaC* are the contents of aromatics, resin, asphaltenes and saturates, respectively.

### 2.7. Micro-Morphology Characterization

Atomic force microscopy (AFM) (BRUKER, New York, NY, USA) that is capable of measuring topographic features at nanometer-scale or even at atomic-scale resolution allows one to visualize precise details of surface topography of bitumen without special sample preparation [33]. In this paper, the AFM imaging was applied to characterize the microstructure of bitumen.

To obtain the samples for AFM observation, 1~1.5 g bitumen heated to 150 °C was laid on a steel disk with the size of 10 mm × 10 mm × 1 mm, and then self-cooled to ambient temperature (about 25 °C). All samples were stored in a container with a glass cap to prevent dust pick-up. The AFM images were observed after the samples were annealed for a minimum of 24 h. The topographic images were scanned in tapping mode with an etched silicon probe. The cantilever was 125 μm long with the curvature radius of 5–10 nm. The drive frequency and amplitude were 260 kHz and 56 mW, respectively. The scan rate was 0.8 Hz. The AFM images showed a 15 μm × 15 μm region.

## 3. Results and Discussions

### 3.1. Characterization of TA and PA

Table 3 summaries the physical properties and chemical components of the TA and PA. Compared with virgin bitumen, the aged bitumen TA and PA exhibit lower penetration, ductility and aromatics content, but higher softening point, viscosity and asphaltenes content, which coincides with the results in previous studies.

It can be also observed from Table 3 that the PI of TA and PA are 1.7 and 3.5, indicate that the colloidal structure of TA and PA are Sol-Gel and Gel respectively, which manifests that the PAV aging influences the bitumen more severely and leads to colloidal structure transformation.

### 3.2. Physical Properties of Regenerated Bitumen

**Figure 1:**
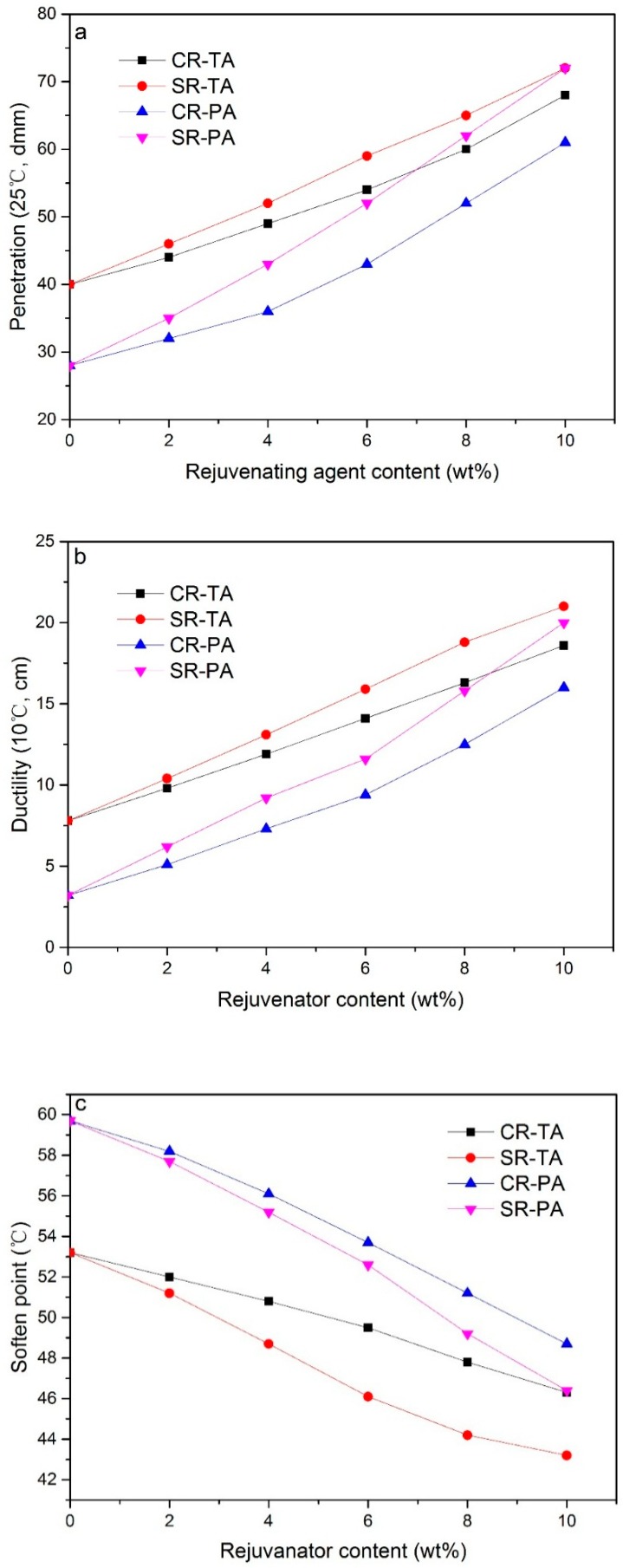

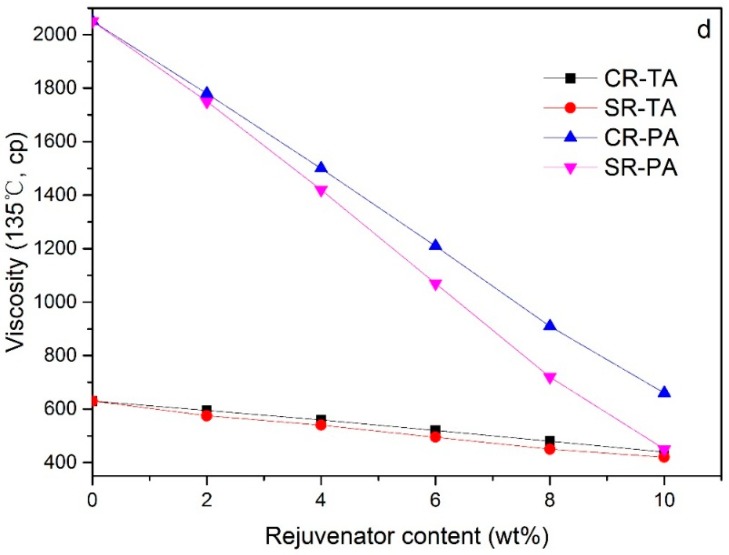
Physical properties of regenerated bitumen with different rejuvenators: (**a**) penetration, (**b**) ductility, (**c**) softening point, (**d**) viscosity.

Figure 1a–d reveal the physical properties of regenerated TA and PA with different contents of CR and SR. It can be observed from the figure that the addition of CR and SR increases the penetration and ductility while decreases the softening point and viscosity.

Compared with CR, SR has more significant influence on the regeneration of aged bitumen. As the most important property of neat bitumen, the penetration of aged bitumen enhances rapidly with the increasing in SR content, comparatively, the influence of the CR content is more moderate. It can be seen from Figure 1a that the regenerated PA with 8 wt % SR owns a penetration of 62 dmm, which meets the requirement of regeneration, whereas the penetration of regenerated PA with 8 wt % CR is only 52 dmm, which is far from the requirement of regeneration. With continuous increasing in contents of SR and CR, the regenerated PA with 10 wt % SR owns a penetration of 72 dmm which is close to that of virgin bitumen, whereas the regenerated PA containing 10 wt % CR merely owns a penetration of 61 dmm which is merely 80 % of virgin bitumen. 

The changing of ductility is similar to that of penetration, as shown in Figure 1b, the ductility increases lineally with the increasing in rejuvenator content, and the ductility of regenerated bitumen with SR is higher than that of regenerated bitumen with CR, the ductility of regenerated PA with 10 wt % SR can be extended from 3.2 cm to 20 cm, which is approximate that of virgin bitumen, whereas the value is 16 cm for regenerated PA with 10 wt % CR.

The softening point and viscosity show an opposite changing to that of penetration and ductility with the addition of rejuvenator. The softening point and viscosity are decreased with the increasing in rejuvenator content. As shown by Figure 1c,d, the SR also influences the softening point and viscosity more significantly than CR, regenerated bitumen with SR shows lower softening point and viscosity than regenerated bitumen with CR at the same rejuvenator content.

It can be also found from Figure 1 that 8 wt % CR and SR can effectively regenerate the physical properties of TA to satisfy the regeneration requirement. With regard to PA, the necessary content of SR for regenerating PA to satisfy the regeneration requirement is 10 wt %, but even if the content of CR reaches up to 10 wt %, the physical properties of PA cannot meet the regeneration requirement.

The result can be ascribed to that CR merely owns high proportion of aromatics, it can only regenerate aged bitumen by replenishing aromatics but can hardly restore the colloidal structure, therefore CR can effectively regenerate TA which maintains the same colloidal structure as virgin bitumen, whereas with regard to PA, its colloidal structure has transformed from Sol-Gel to Gel, thus CR cannot effectively regenerate the physical properties of PA due to its definite influence on colloidal structure restoration. Compared with CR, SR contains both high proportion of aromatics and sulfonic group, it can replenishing PA with necessary aromatics and react with the superficial atoms of asphaltenes to reform the solvation layer covering the surface of asphaltenes, which facilitates gathered asphaltenes stably disperse in colloidal solution at a smaller size and contributes the colloidal structure restoration of PA, therefore SR can regenerate PA more effectively than CR.

### 3.3. Chemical Components of Regenerated Bitumen

Table 4 and Table 5 show the chemical components of regenerated bitumen containing different contents of CR and SR. It can be found from that the Tables that with the increasing in contents of CR and SR, the aromatics content of regenerated asphaltenes increases, whereas the asphaltenes content declines, compared with relatively regular changing of aromatics content and asphaltenes content, the resins content and saturates contents changed unregularly with the increasing in rejuvenator contents.

It can be found from Table 4 and Table 5 that the chemical components of regenerated bitumen containing SR and CR are similar at the same rejuvenator content, which indicates that the SR and CR influence the chemical components reconstitution of TA and PA similarly.

### 3.4. Colloidal Structure Stability of Regenerated Bitumen

Aging changes the chemical components of bitumen, and then negatively influence the compatibility between chemical components, which declines the colloidal structure stability of bitumen, therefore the regeneration effect of rejuvenator on aged bitumen can be investigated by characterization of colloidal structure stability index (CSI).

**Figure 2:**
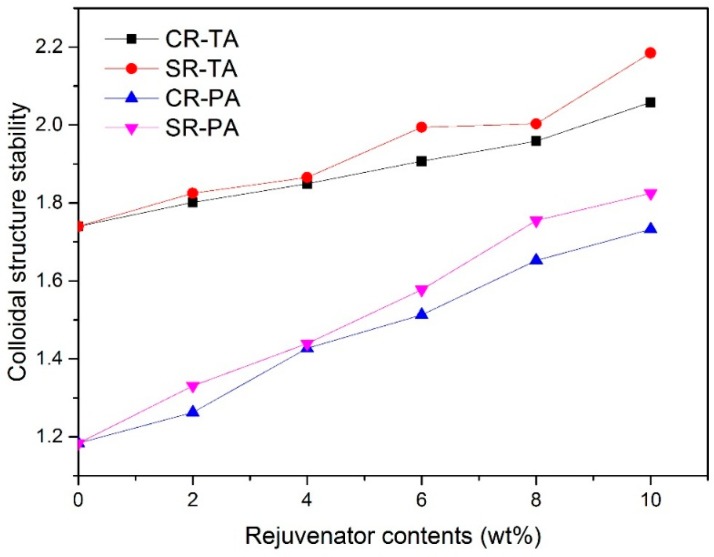
CSI of regenerated bitumen with different rejuvenators.

Figure 2 reveals the CSI of regenerated bitumen with different contents of CR and SR. It can be observed from Figure 2 that, the CSI of regenerated bitumen increase with the addition of rejuvenator, which indicates that the colloidal structure stability of aged bitumen can be improved by rejuvenator.

As described by Figure 2, regenerated bitumen containing SR displays high CSI than regenerated bitumen containing CR at the same rejuvenator content, for instance, 10 wt % SR and CR can regenerate the CSI of TA by increasing from 1.74 for TA to 2.03 and 2.27, respectively, which indicates that SR can enhance the CSI of aged bitumen more significantly than CR.

The result mentioned above can be explained as following: The CR contain high proportion of aromatics and low proportion of resins and asphaltenes (presented in Table 2), it can only improve the colloidal structure stability by replenishing aromatics to dilute content of asphaltenes, comparatively, the SR contains both high proportion of aromatics and active sulfonic group, it can on the one hand replenish aromatics to reconstitute the chemical components of aged bitumen, and on the other hand can reform the solvation layer covering the surface of asphaltenes by sulfonic group reacting with the superficial atoms of asphaltenes in aged bitumen, therefore SR can improves the colloidal structure stability more effectively than CR.

### 3.5. Colloidal Structure Type of Regenerated Bitumen

**Figure 3:**
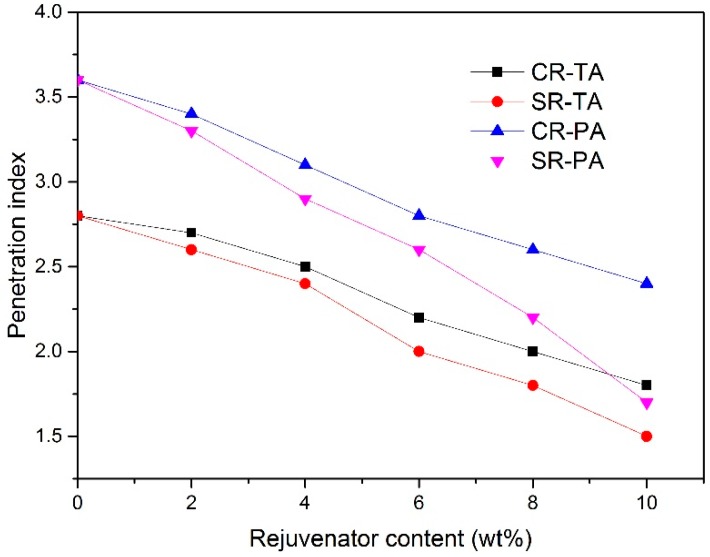
PI of regenerated bitumen with different rejuvenators.

Figure 3 shows the PI of regenerated bitumen with different contents of CR and SR. It can be observed from Figure 3 that, the PI of regenerated bitumen declines with the addition of rejuvenator, as illustrated in Figure 3, 10 wt % SR and CR can decrease the PI from 2.8 for TA to 1.5 and 1.8, indicating that the colloidal structure of TA has transformed from Gel to Sol-Gel, which implies that both CR and SR equally influence the regeneration of TA, this result is coincident with the physical properties results of regenerated TA containing SR and CR.

It can be also observed from Figure 3 that the PI of regenerated PA containing SR is lower than that of regenerated PA containing CR at the same rejuvenator content, for example, 10 wt % SR can decrease the PI from 3.6 for PA to 1.7, indicating that the colloidal structure of regenerated PA containing 10 wt % SR has transformed from Gel to Sol-Gel, nevertheless the PI for regenerated PA containing 10 wt % CR is 2.4, indicating that the regenerated PA containing 10 wt % CR maintains Gel structure.

The result can be also ascribed to the chemical interaction between asphaltenes and SR. The active sulfonic group in SR can react with the superficial atoms of asphaltenes in PA, and then a solvation layer forms and covers on the surfaces of asphaltenes, which facilitate the asphaltenes to be uniformly re-dispersed in colloidal solution and contributes to the colloidal structure transformation from Gel to Sol-Gel. Compared with SR, CR can only replenish PA with aromatics, which can barely facilitate the colloidal structure transformation of PA, thus the regenerated PA maintains a colloidal structure of Gel even if the CR content reaches up to 10 wt %.

### 3.6. AFM Characterization

The bitumen displays a bee-like structure which can be observed by AFM images, and the dimension of the bee-like structures is increased with the asphaltenes precipitating from the other chemical components. As is known, the colloidal structure is subjected to be damaged by aging, which results in the asphaltenes precipitating from the other chemical components and increasing in dimension of the bee-like structures.

It can be inferred from the physical properties testing results in this paper that the SR can reconstitute the colloidal structure more effectively than CR, which was confirm by penetration index testing results. In an effort to further assess the effect of CR and SR on colloidal structure restoration of different aged bitumen, AFM testing was performed on at multiple locations across the virgin bitumen, aged bitumen and regenerated bitumen.

**Figure 4:**
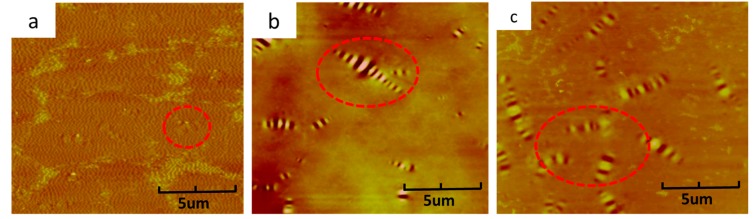
AFM images of virgin bitumen, TFOT aged bitumen and PAV aged bitumen: (**a**) virgin bitumen, (**b**) TFOT aged bitumen, (**c**) PAV aged bitumen.

Figure 4a–c show the surface micro-morphology of virgin bitumen, TFOT aged bitumen and PAV aged bitumen. It can be observed from Figure 4a that the surface of virgin bitumen is even and only a little bee-like structure is existed. However, the surface becomes rough after aging, which can be attributed to the damage of colloidal structure caused by aging, as shown in Figure 4b,c. In comparison with TFOT aged bitumen, PAV aged bitumen shows a rougher surface than TFOT aged bitumen, indicating that PAV aging damages the colloidal structure of virgin bitumen more severely than TFOT aging.

**Figure 5:**
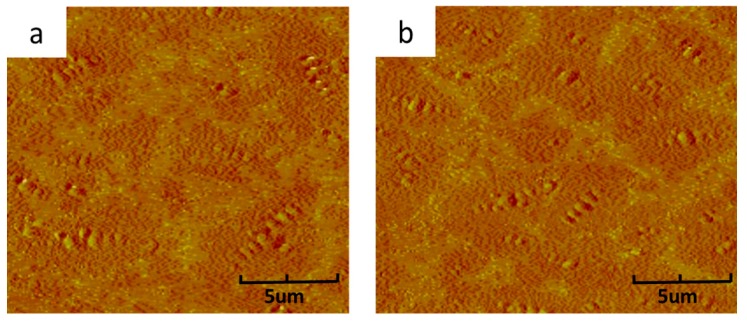
AFM images of regenerated TFOT aged bitumen: (**a**) regenerated TFOT aged bitumen with 10 wt % CR, (**b**) regenerated TFOT aged bitumen with 10 wt % SR.

The surface micro-morphology of regenerated TFOT aged bitumen by 10 wt % CR and SR is described in Figure 5a,b. It can be seen from Figure 5 that the surface of TFOT aged bitumen become smooth with introduction of 10 wt % CR and SR. Besides, it can be also observed that the surface micro-morphology of regenerated TFOT aged bitumen by 10 wt % CR share similarities with that of regenerated TFOT aged bitumen by 10 wt % SR, indicating that CR reconstitutes the colloidal structure of TFOT aged bitumen equally to SR, which is consistent with the physical properties and penetration index testing results of regenerated TFOT aged bitumen containing CR and SR.

**Figure 6:**
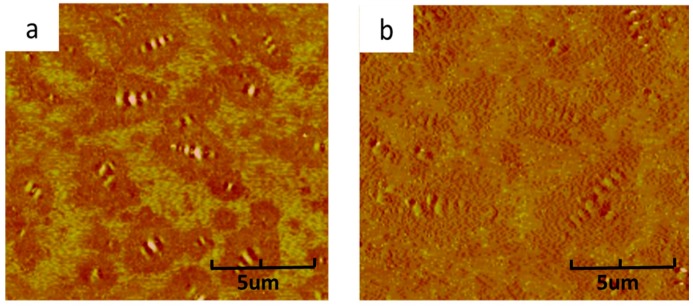
AFM images of regenerated PAV aged bitumen: (**a**) regenerated PAV aged bitumen with 10 wt % CR, (**b**) regenerated PAV aged bitumen with 10 wt % SR.

Figure 6a,b demonstrate the micro-morphology of regenerated PAV aged bitumen by 10 wt % CR and SR. It can be found by comparing Figure 6a and Figure 4c that, the regenerated PAV aged bitumen by 10 wt % CR maintains nearly the same surface micro-morphology as PAV aged bitumen, indicating that the CR can barely decrease the dimension of bee-like structure, however, the surface micro-morphology of regenerated PAV aged bitumen by 10 wt % SR shows similarities with that of virgin bitumen before aging, indicating that the SR can effectively facilitate the re-dispersion of asphaltenes of PAV aged bitumen, which contributes to the colloidal structure restoration of PAV aged bitumen and performance improvement, this results is also in good agreement with the physical properties and penetration index testing results of regenerated PAV aged bitumen containing CR and SR.

## 4. Conclusions

DBSA was adopted as a solubilizer together with conventional rejuvenator (CR) to produce a solubilized rejuvenator (SR). Effect of SR and CR on the physical properties, colloidal structure and micro-morphology of TFOT aged bitumen and PAV aged bitumen were investigated. The conclusions were as follows:

1. CR and SR influenced the chemical components of TFOT aged bitumen and PAV aged bitumen similarly, thus for TFOT aged bitumen that its physical properties attenuation were mainly attributed to the chemical component variation, can be effectively regenerated by 8 wt % of CR and SR. The physical properties of regenerated TFOT aged bitumen with 8 wt % of CR and SR can approximate to that of virgin bitumen before aging.

2. SR can replenish PAV aged bitumen with necessary amounts of aromatics and reconstitute the micellar nucleus dispersion in colloidal structure of PAV aged bitumen simultaneously, thus the physical properties of PAV aged bitumen can be recovered to the condition of virgin bitumen by 10 wt % of SR, comparatively, CR can barely influence the colloidal structure transformation of PAV aged bitumen, therefore the physical properties of PAV aged bitumen with 10 wt % of CR cannot meet the regeneration requirement yet.

3. The sulfonic group in DBSA can react with superficial atoms of asphaltenes to reform a solvation layer covering on the surface of asphaltenes, which contributes to colloidal structure transformation of aged bitumen, therefore PAV aged bitumen can be recovered from Gel to Sol-Gel by 10 wt % SR, and the dimension of beelike structure formed by asphaltenes can be approximate that of virgin bitumen.

Further research is required to confirm practical application and possibilities of DBSA incorporating with conventional rejuvenator road construction as well as the difference of regenerating effect on aged bitumen compared to conventional rejuvenator. For example, the road performance of recycled hot mix asphalt containing CR and SR as well as some other rejuvenators should be comparatively investigated.

## Figures and Tables

**Table 1:** Physical properties and chemical components of virgin bitumen.

Items		Value
Physical properties	Softening point (°C)	45.2
	Penetration (25 °C, dmm)	74
	Ductility (10 °C, cm)	22
	Viscosity (135 °C, cp)	420
Colloidal structure	Penetration index ^a^	1.2
Chemical components	Saturates (%)	15.3
	Aromatics (%)	44.6
	Resins (%)	28.8
	Asphaltenes (%)	11.3

^a^ Penetration index was calculated according to [26].

**Table 2:** Physical properties and chemical components of SR and CR.

Items		SR	CR
Physical properties	Flash point (°C)	>220	>220
	Viscosity (60 °C, Pa·s)	0.95	1.52
	Viscosity ratio after TFOT	1.7	2.1
	Weight loss after TFOT (%)	−1.5	−2.2
Chemical components	Saturates (%)	13.3	12.5
	Aromatics (%)	62.7	64.2
	Resins (%)	17.3	15.4
	Asphaltenes (%)	6.7	7.9

**Table 3:** Physical properties and chemical components of TA and PA.

Items		TA	PA
Physical properties	Softening point (°C)	53.2	59.7
	Penetration (25 °C, dmm)	40	28
	Ductility (10 °C, cm)	7.8	3.2
	Viscosity (135 °C, cp)	630	2050
Colloidal structure	Penetration index	1.7	3.5
Chemical components	Saturates (%)	14.5	12.4
	Aromatics (%)	37.3	29.6
	Resins (%)	26.2	24.6
	Asphaltenes (%)	22.0	33.4

**Table 4:** Chemical components of regenerated TA containing CR and SR.

Samples	Rejuvenator Content (wt %)	Weight of Chemical Components (wt %)			
		Sa	Ar	Re	As
CR-TA	0	14.5	37.3	26.2	22
	2	14.2	38.5	25.8	21.5
	4	14.4	38.9	26	20.7
	6	14.6	39.7	25.9	19.8
	8	15.4	40.7	25.5	18.4
	10	15.2	41.7	25.6	17.5
SR-TA	0	14.5	37.3	26.2	22
	2	14.1	38.1	26.5	21.3
	4	14.5	40.4	24.7	20.4
	6	14.2	41.9	24.7	19.2
	8	14.4	42.3	24.4	18.9
	10	14.1	43.5	25.1	17.3

**Table 5:** Chemical components of regenerated PA containing CR and SR.

Samples	Rejuvenator Content (wt %)	Weight of Chemical Components (wt %)			
		Sa	Ar	Re	As
CR-PA	0	12.4	29.6	24.6	33.4
	2	11.8	31.3	24.5	32.4
	4	12.6	34.6	24.2	28.6
	6	12.5	36.4	23.8	27.3
	8	12.5	38.2	24.1	25.2
	10	13.1	40	23.4	23.5
SR-PA	0	12.4	29.6	24.6	33.4
	2	11.7	31.3	25.8	31.2
	4	11.4	33.7	25.3	29.6
	6	12.5	37.9	23.3	26.3
	8	12.2	39.8	23.9	24.1
	10	12.9	41	23.6	22.5

## References

[1] Elkashef M., Williams R., Cochran E. (2018). Physical and chemical characterization of rejuvenated reclaimed asphalt pavement (RAP) binders using rheology testing and pyrolysis gas chromatography-mass spectrometry. Mater. Struct..

[2] Zhang K., Muhunthan B. (2017). Effects of production stages on blending and mechanical properties of asphalt mixtures with reclaimed asphalt pavement. Constr. Build. Mater..

[3] Gao L., Li H., Xie J., Yu Z. (2018). Evaluation of pavement performance for reclaimed asphalt materials in different layers. Constr. Build. Mater..

[4] Vegazamanillo A., Calzadaperez M., Lastragonzalez P., Indacoecheavega I., Fernandezortega J.A. (2017). Analysis of the use of cupola furnace slags, green sand and reclaimed asphalt pavement in asphalt concrete mixtures for low intensity traffic. Revista de la Construccion.

[5] Chen M., Leng B., Wu S., Sang Y. (2014). Physical, chemical and rheological properties of waste edible vegetable oil rejuvenated asphalt binders. Constr. Build. Mater..

[6] Shu X., Huang B., Vukosavljevic D. (2008). Laboratory evaluation of fatigue characteristics of recycled asphalt mixture. Constr. Build. Mater..

[7] Sivilevicius H., Braziunas J., Prentkovskis O. (2017). Technologies and principles of hot recycling and investigation of preheated reclaimed asphalt pavement batching process in an asphalt mixing plant. Appl. Sci..

[8] Vislavicius K., Sivilevicius H. (2013). Effect of reclaimed asphalt pavement gradation variation on the homogeneity of recycled hot-mix asphalt. Arch. Civ. Mech. Eng..

[9] Xu J., Hao P., Zhang D., Yuan G. (2018). Investigation of reclaimed asphalt pavement blending efficiency based on micro-mechanical properties of layered asphalt binders. Constr. Build. Mater..

[10] Xiao Y., Li C., Wan M., Zhou X., Wang Y., Wu S. (2017). Study of the diffusion of rejuvenators and its effect on aged bitumen binder. Appl. Sci..

[11] Arshad M., Ahmed M. (2017). Potential use of reclaimed asphalt pavement and recycled concrete aggregate in base/subbase layers of flexible pavements. Constr. Build. Mater..

[12] Yang S., Lee L. (2016). Characterizing the chemical and rheological properties of severely aged reclaimed asphalt pavement materials with high recycling rate. Constr. Build. Mater..

[13] Kuang D., Feng Z., Yu J. (2011). A new approach for evaluating rejuvenator diffusing into aged bitumen. J. Wuhan Univ. Technol..

[14] Cavalli M., Zaumanis M., Mazza E., Partl M., Poulikakos L. (2018). Effect of ageing on the mechanical and chemical properties of binder from RAP treated with bio-based rejuvenators. Compos. Part B.

[15] Gamarra A., Ossa E. (2018). Thermo-oxidative aging of bitumen. Int. J. Pavement Eng..

[16] Mangiafico S., Benedetto H., Sauzeat C., Olard F., Pouget S., Planque L. (2016). Effect of colloidal structure of bituminous binder blends on linear viscoelastic behaviour of mixtures containing reclaimed asphalt pavement. Mater. Des..

[17] Zhang H., Wang H., Yu J. (2011). Effect of aging on morphology of organo-montmorillonite modified bitumen by atomic force microscopy. J. Microsc..

[18] Jahromi S., Khodaii A. (2009). Effects of nanoclay on rheological properties of bitumen binder. Constr. Build. Mater..

[19] Loeber L., Sutton O., Morel J., Valleton J., Muller G. (2010). New direct observations of asphalts and asphalt binder by scanning electron microscopy and atomic force microscopy. J. Microsc..

[20] Baumgardner G., Masson J., Hardee J., Menapace A., Williams A. (2006). Polyphosphoric acid modified asphalt: Proposed mechanisms. J. Assoc. Asph. Paving Technol..

[21] Kuang D., Yu J., Cai Z. (2011). Effect of rejuvenator on rejuvenation properties of aged bitumen of different aging degree. Highway.

[22] Lee S., Amirkhanian S., Park N., Kim K. (2007). Characterization of warm mix asphalt binder containing artifically long-term aged binders. Constr. Build. Mater..

[23] Shen J., Amirkhanian S., Miller J. (2007). Effects of rejuvenating agents on superpave mixtures containing reclaimed asphalt pavement. J. Mater. Civ. Eng..

[24] Kuang D. (2012). Preparation of diffusible rejuvenator and its influence on performances of recycled bitumen and recycled asphalt mixture. Ph.D. Thesis.

[25] Zargar G., Gheysari R., Takassi M., Rostami A., Zadehnazari A. (2018). Evaluation of a sulfanilic acid based surfactant in crude oil demulsification: An experimental study. Oil Gas Sci. Technol..

[26] ASTM D 5 (2005). Standard Test Method for Penetration of Bituminous Materials.

[27] ASTM D 1754 (2014). Standard Test Method for Effects of Heat and Air on Asphaltic Materials.

[28] ASTM D 6521 (2013). Standard Practice for Accelerated Aging of Asphalt Binder Using a Pressurized Aging Vessel Lpar.

[29] ASTM D 36 (2006). Standard Test Method for Softening Point of Bitumen.

[30] ASTM D 113 (2016). Standard Test Method for Ductility of Bituminous Materials.

[31] ASTM D 113 (2012). Standard Test Method for Viscosity Determination of Asphalt at Elevated Temperatures Using a Rotational Viscometer.

[32] ASTM D 4124 (2018). Standard Test Method for Separation of Asphalt into Four Fractions.

[33] Yu X., Burnham N., Tao M. (2015). Surface microstructure of bitumen characterized by atomic force microscopy. Adv. Colloid Interface Sci..

